# Intracochlear Recordings of Acoustically and Electrically Evoked Potentials in Nucleus Hybrid L24 Cochlear Implant Users and Their Relationship to Speech Perception

**DOI:** 10.3389/fnins.2017.00216

**Published:** 2017-04-19

**Authors:** Jae-Ryong Kim, Viral D. Tejani, Paul J. Abbas, Carolyn J. Brown

**Affiliations:** ^1^Department of Communication Sciences and Disorders, University of IowaIowa City, IA, USA; ^2^Department of Otolaryngology-Head and Neck Surgery, Inje University College of MedicineBusan, South Korea; ^3^Department of Otolaryngology-Head and Neck Surgery, University of Iowa Hospitals and ClinicsIowa City, IA, USA

**Keywords:** cochlear implant, auditory evoked potentials, electrocochleography, electrically evoked compound action potential, hybrid cochlear implant, neural response telemetry

## Abstract

The Hybrid cochlear implant (CI) has been developed for individuals with high frequency hearing loss who retain good low frequency hearing. Outcomes have been encouraging but individual variability is high; the health of the cochlea and the auditory nerve may be important factors driving outcomes. Electrically evoked compound action potentials (ECAPs) reflect the response of the auditory nerve to electrical stimulation while electrocochleography (ECochG) reflects the response of the cochlear hair cells and auditory nerve to acoustic stimulation. In this study both ECAPs and ECochG responses were recorded from Nucleus Hybrid L24 CI users. Correlations between these two measures of peripheral auditory function and speech perception are reported. This retrospective study includes data from 25 L24 CI users. ECAPs and ECochG responses were recorded from an intracochlear electrode using stimuli presented at or near maximum acceptable loudness levels. Speech perception was assessed using Consonant-Nucleus-Consonant (CNC) word lists presented in quiet and AzBio sentences presented at a +5 dB signal-to-noise ratio in both the combined acoustic and electric (A+E) and electric (E) alone listening modes. Acoustic gain was calculated by subtracting these two scores. Correlations between these physiologic and speech perception measures were then computed. ECAP amplitudes recorded from the most apical electrode were significantly correlated with CNC scores measured in the E alone (*r* = 0.56) and A+E conditions (*r* = 0.64), but not with performance on the AzBio test. ECochG responses recorded using the most apical electrode in the intracochlear array but evoked using a 500 Hz tone burst were not correlated with either the scores on the CNC or AzBio tests. However, ECochG amplitude was correlated with a composite metric relating the additional benefit of acoustic gain in noise relative to quiet conditions (*r* = 0.67). Both measures can be recorded from Hybrid L24 CI users and both ECAP and ECochG measures may result in more complete characterization of speech perception outcomes than either measure alone.

## Introduction

Since cochlear implants (CIs) were first introduced into clinical practice in the mid-1980s, CI technology has changed significantly. Those changes led to marked improvements in performance and today, CIs are considered to be the treatment of choice for individuals with bilateral profound sensorineural hearing loss (SNHL). Recently, and based in large part on the positive outcomes exhibited by standard CI users, candidacy criteria have been relaxed to include individuals with good low frequency hearing but severe-to-profound high frequency SNHL (Cohen, 2004; Lenarz et al., 2013; Roland et al., 2016). Hearing aids often provide only limited benefit for this population (Hornsby and Ricketts, 2006; Turner, 2006) making CIs an attractive alternative. However, insertion trauma associated with implanting a standard long electrode array often resulted in complete loss of residual acoustic hearing in the implanted ear. Hybrid CIs were developed specifically for this population and designed to help preserve residual acoustic hearing in the implanted ear (Gantz and Turner, 2003; Lenarz et al., 2009).

The original S8 Hybrid CI was manufactured by Cochlear Ltd. for investigational purposes and had a shorter electrode array (10 mm) and fewer intracochlear electrodes (6 electrodes) than the standard, long 22-electrode arrays offered by Cochlear Ltd. (Gantz and Turner, 2003). The goal was for the intracochlear electrode array to be inserted into the cochlea without adversely affecting residual low frequency acoustic hearing. Low frequency sounds were intended to be processed normally (with or occasionally without amplification). High frequency sounds were transmitted electrically, bypassing the damaged cochlear hair cells and stimulating the auditory nerve directly (Turner et al., 2008a). Preliminary results were promising (Turner et al., 2004; Gantz et al., 2009; Woodson et al., 2010). On average, speech perception scores measured in quiet and in background noise were significantly better when the listeners were allowed to combine both acoustic and electrical (A+E) input compared to when they were tested using either in the acoustic (A) alone or electrical (E) alone listening modes. Additionally, speech perception in noise was better for S8 Hybrid users compared to the standard 22-electrode implant users (Turner et al., 2004, 2008b). These findings led to the development of the commercially released Nucleus L24 Hybrid electrode array (described in more detail in “Materials and Methods”). Studies again showed good performance (Büchner et al., 2009; Lenarz et al., 2009, 2013; Roland et al., 2016), but individual variability remains high. Some Hybrid CI users (regardless of manufacturer and length of array) benefited tremendously from having access to both acoustic and electrical signals, while others did not (Kiefer et al., 2005; Reiss et al., 2008; Lenarz et al., 2013; Gantz et al., 2016; Roland et al., 2016).

Outcomes with a CI are a result of multiple factors (Lazard et al., 2012; Blamey et al., 2013; Holden et al., 2013; Shearer et al., 2017). Recent investigations have suggested that better outcomes with a traditional or Hybrid CI might be expected from individuals presenting with better overall “cochlear health” (Gantz et al., 2009; Kim et al., 2010; Fitzpatrick et al., 2013; Formeister et al., 2015). In other words, CI candidates who present with better hair cell and/or neural survival may have better outcomes. Cochlear health might be more important for Hybrid candidates with residual hearing than for traditional CI candidates. In this study, we use the Neural Response Telemetry (NRT) system to measure the response of the peripheral auditory system to both acoustic and electrical stimulation. Our goal is to explore the relationship between these objective measures of the status of the auditory periphery and speech perception.

Electrically evoked compound action potentials (ECAPs) are recordings of the synchronous response from a large number of auditory nerve fibers to the presentation of a brief electrical impulse. They are characterized by a negative peak (N1) that is recorded approximately 0.2–0.4 ms following the onset of the stimulus and is followed by a positive peak (P2) at 0.6–0.8 ms (Brown et al., 1998, 2000; Abbas et al., 1999). ECAPs are recorded routinely following cochlear implantation and do not require the presence of viable cochlear hair cells. As early as 1958, Goldstein and Kiang theorized that the amplitude of neural potentials should increase as the number of active neurons increased. Animal studies later showed that electrically evoked neural potentials are correlated with neural survival (Smith and Simmons, 1983; Hall, 1990; Miller et al., 1994; Prado-Guitierrez et al., 2006). One may theorize that stronger ECAPs or greater neural survival would reflect better CI outcomes (Kim et al., 2010; Seyyedi et al., 2014) but this has been somewhat difficult to prove. Kim et al. (2010) reported finding correlations between the slope of the ECAP amplitude growth functions and performance. That study included subjects who used both older generation devices (Nucleus CI24M standard implant and the 24M S8 Hybrid implant) and newer technology (Nucleus 24RE standard implant and the 24RE S8 Hybrid implant). The major difference between the older and newer implants was the lower noise floor of the amplifier on the newer devices. The noise floor of the measurement system could impact slope of the ECAP growth functions. Kim et al. (2010) reported that the slope of the ECAP growth functions measured using the newer technology implants was correlated with performance. This was not the case for the older generation of CIs.

Acoustically evoked neural responses can also be recorded from the auditory periphery. This measure is typically referred to as an electrocochleography (ECochG). ECochGs have traditionally been recorded using an electrode placed on the tympanic membrane or the promontory of the middle ear. They have played a role in diagnosing Meniere's disease (Gibson et al., 1977) and more recently have been used to explore the pathophysiology of a condition often described as “hidden hearing loss” where audiometric thresholds are normal but patients struggle to understand speech in background noise (Liberman et al., 2016). ECochGs have also been recorded using a round window electrode from individuals undergoing CI surgery. High level acoustic tone bursts that range in frequency from 250 to 4,000 Hz were presented. These responses were combined offline to generate a metric called the “total cochlear response” (Fitzpatrick et al., 2013; Formeister et al., 2015). Importantly, Fitzpatrick et al. (2013) and Formeister et al. (2015) reported a significant correlation between the magnitude of the ongoing ECochG response across several frequencies and postoperative speech perception in adults and children using standard CIs.

Recently, several researchers described ECochG recordings obtained from CI users with residual hearing during the post-operative period (Dalbert et al., 2015; Abbas et al., 2017; Koka et al., 2017). Across these studies, acoustic stimuli were presented and ECochG recordings were obtained from an intracochlear electrode. Koka et al. (2017) and Abbas et al. (2017) used recording and analysis methods to emphasize contributions from either the cochlear hair cells or the auditory nerve. Significant correlations between the ECochG responses and audiometric thresholds were also reported. Results showed that acoustically generated ECochG responses could be used to monitor changes in hearing status following cochlear implantation.

In this study we propose to use a combination of both acoustic and electrical stimulation to more fully characterize the status of the auditory periphery in Hybrid L24 CI users. We argue that the two measures should provide a more complete profile of the status of the peripheral auditory system than either measure individually. Our goal is to determine the extent to which ECAP responses, which likely reflect the response primarily from the relatively basal region of the cochlea to electrical stimulation, and the acoustically evoked ECochG responses, which provide a measure of hair cell and neural responses from more apical regions of the cochlea, might be combined to more accurately characterize the status of the auditory periphery. We compare these measures to speech perception results obtained from a group of Hybrid L24 CI users to test the hypothesis that speech perception is related to the status of the auditory periphery. More specifically, we will assess if individuals with more robust (e.g., largest) ECAPs will exhibit better performance when testing is conducted in the electric only listening mode than individuals who have smaller amplitude ECAP responses. Additionally, we will assess if Hybrid CI recipients who enjoy the most benefit from use of acoustic stimulation are those who also present with the most robust (e.g., largest) acoustically evoked ECochG responses.

## Materials and methods

This was a retrospective study. Records from individuals who received a Nucleus Hybrid L24 CI at the University of Iowa Hospitals and Clinics between 2010 and 2015 were reviewed and information about speech perception extracted. These results were then compared with ECAP and ECochG data also collected in our lab. The ECochG data was recently published (Abbas et al., 2017). That report focused on describing analysis techniques to emphasize contributions of hair cells and the auditory nerve to the ECochG response. In this report we focus on an alternative measure of ECochG magnitude. We also include measures of neural response to an electrical stimulus (ECAP) that were not included in the Abbas et al. (2017) study. All of the procedures used in this study were approved by the University of Iowa Institutional Review Board (IRB) and all subjects gave written informed consent in accordance with the Declaration of Helsinki.

### Nucleus hybrid L24 CI

The Nucleus Hybrid L24 CI is manufactured by Cochlear Ltd. The internal electrode array is 16 mm in total length and contains 22 electrode contacts. It is thinner than the previous generation CI24RE CI, and the electrode array is designed to rest against the lateral wall of the cochlea. The implanted electrode array spans approximately 270° of the basal turn of the cochlea with the most apical electrode lying at a place thought to correspond to approximately 1,500–2,000 Hz (Greenwood, 1990; Stakhovskaya et al., 2007; Lenarz et al., 2009, 2013; Jurawitz et al., 2014; Roland et al., 2016). The L24 array was approved by the Food and Drug Administration (FDA) for clinical use in March 2014; arrays implanted prior to that date were implanted under an FDA Investigational Device Exemption (IDE) status (IDE G070191 and G110089). The external processor used with this device includes both an electrical and acoustic component. It is designed to allow the user to integrate electric and acoustic information simultaneously and can be programmed to accommodate the extent and configuration of a recipient's acoustic hearing following surgery.

### Subjects

**Figure 1:**
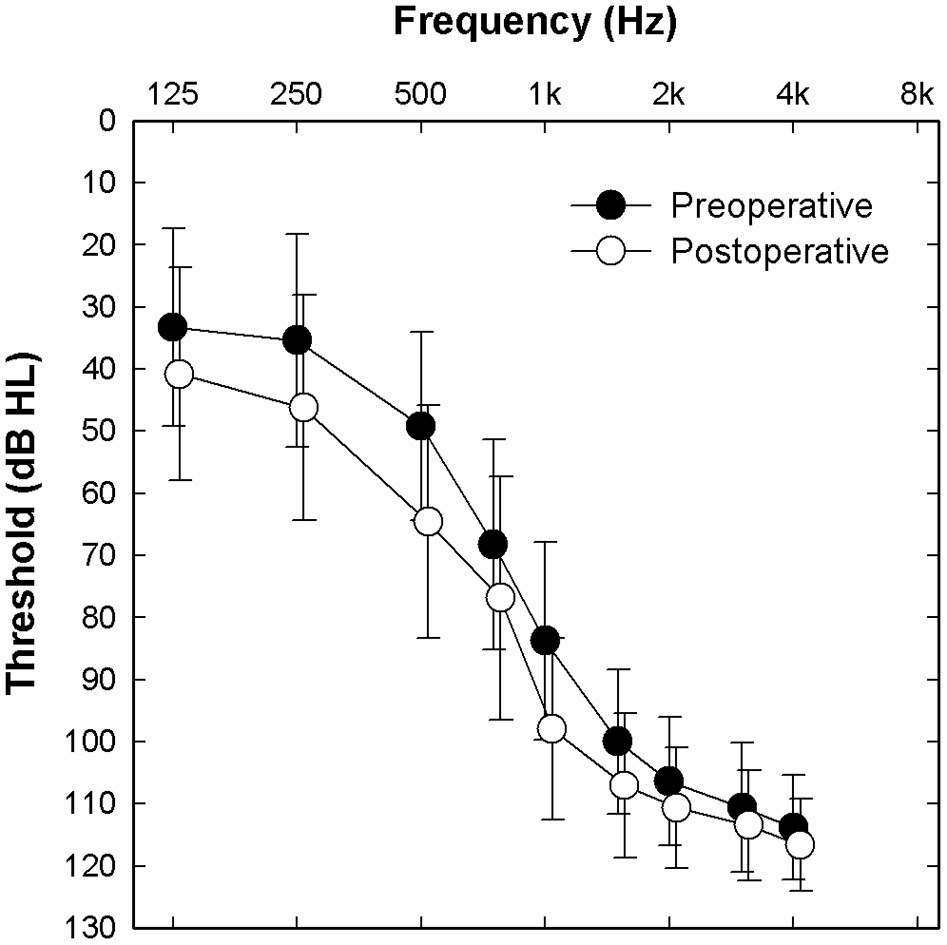
**Mean pre- and post-operative audiometric thresholds for the implanted ear of all 25 study participants**. Error bars indicate ± 1 standard deviation.

Twenty-five adult Nucleus Hybrid L24 CI users participated in this study. Table 1 shows demographic information about the study participants. Forty-four percent were male. Fifty-six percent were female. Approximately equal numbers of right and left ears were implanted. For the majority of study participants, the etiology of their hearing loss was unknown. Subjects ranged in age from 18 to 65 years at the time of surgery. Mean duration of hearing loss prior to CI surgery was 28 years (*SD* = 16 years) and mean duration of hearing aid use was 17 years (*SD* = 12 years). Preoperative Consonant-Nucleus-Consonant (CNC) word scores were, on average, 22% correct. Though for the purposes of this report, it was not necessary to compare preoperative and postoperative audiometric thresholds, we included this data for informative purposes (Figure 1). Postoperative thresholds were measured at the time ECAP, ECochG, and speech perception data were obtained. The majority of study participants had low frequency acoustic hearing (pure tone average of 250, 500, and 1,000 Hz) within 15 dB of their preoperative pure tone thresholds. Three subjects lost significant amounts of acoustic hearing post-operatively (>30 dB) and were also included in this report. Inclusion criteria required that the selected participants had stable residual hearing at the time of evoked potential and speech perception testing since, on occasion, testing occurred at two different points in time. Since ECochG responses in Hybrid users remain stable over time for those with stable residual hearing (Abbas et al., 2017), the different time periods of testing in some subjects was not concerning.

**Table 1:** **Demographic and audiological history for study participants**.

	***n*** **(%)**		**mean ± SD**
Gender		Age at implantation (years)	50 ± 13
Male	11 (44%)		
Female	14 (56%)	Duration of HL (years)	28 ± 16
Ear implanted			
Right	13 (52%)	Duration of HA use (years)	17 ± 12
Left	12 (48%)		
Etiology		Preoperative PTA^§^ (dB HL)	56 ± 13
Unknown	15 (60%)		
Hereditary	4 (16%)	Postoperative PTA^§^ (dB HL)	70 ± 15
Noise exposure	4 (16%)		
Autoimmune	2 (8%)	Preoperative CNC word (%)	22 ± 16

§ *Pure tone average of 0.25, 0.5, 1 kHz (No responses were converted to 120 dB HL)*.

*HL indicates hearing loss; HA, hearing aid; PTA, pure tone average; CNC, consonant-nucleus-consonant; SD, standard deviation*.

The 25 participants were part of a larger pool of individuals with hearing preservation implants who participated in earlier studies in our lab where post-operative ECochG and ECAP data were collected. Subjects were awake during the testing procedures. ECochG growth functions were collected using acoustic 500 Hz tone bursts and recorded using the most apical intracochlear electrode (Abbas et al., 2017). ECAP growth functions were collected from a subset of electrodes spaced across the array. Speech perception scores were extracted from the patient's clinical records. ECAP and ECochG data were generally collected at the same point in time (no earlier than 1 month post activation). Speech perception testing was conducted no earlier than 6 months post activation. All 25 study participants had been fit with and regularly used an acoustic component with their speech processor. The frequency boundary for acoustic-electric stimulation was defined as the highest audiometric frequency with an unaided audiometric threshold less than or equal to 70 dB HL (Cochlear Ltd., 2015). The acoustic component of the Hybrid system was programmed using the NAL-NL2 fitting formula (Keidser et al., 2011). In some instances, acoustic output was modified slightly to address problems with loudness tolerance. Frequencies higher than the acoustic-electric boundary were delivered via electrical stimulation.

### Electrophysiologic recordings: electrical stimulation

ECAPs were recorded using standard clinical software provided by Cochlear Ltd. (Custom Sound EP, version 4.3). Stimuli were biphasic current pulses presented in a monopolar stimulation mode at 80 Hz stimulation rate. Pulse durations were typically 25 μs/phase with a 7 μs interphase gap. Higher pulse durations (37 or 50 μs) were used in some cases to overcome voltage compliance limits. Three stimulating electrodes widely spaced across the electrode array were selected for testing. They included an apical electrode (20, 21, or 22), a middle electrode (12, 13, or 14), and a basal electrode (6, 7, or 8). Typically, an electrode located two electrodes apical relative to the stimulating electrode was used for recording. ECAPs were obtained at a 20 kHz sampling rate using the standard subtraction method detailed elsewhere (Brown et al., 1998, 2000; Abbas et al., 1999). Amplitude growth functions were obtained for these test electrodes. These functions were generated by a series of ECAPs that were recorded at probe levels that varied from just below the uncomfortable loudness level (UCL)—labeled here the maximum comfortable level (MCL) - to below the visual detection threshold. For the purposes of this retrospective review, only the ECAP amplitude recorded at MCL was used for correlational analysis.

ECAP waveforms consisted of an average of approximately 50–100 sweeps, and were analyzed offline using a custom MATLAB script. N1 and P2 peaks were selected manually and the ECAP amplitude for each waveform was defined as the voltage difference between the N1 and P2 peaks.

### Electrophysiologic recordings: acoustic stimulation

Acoustically evoked ECochG responses were recorded using Custom Sound EP (version 3.2). Details of the recording technique have been reported elsewhere (Abbas et al., 2017). Briefly, a research patch allowed Custom Sound EP to trigger an external acoustic stimulus. The stimulus was a 12 ms, 500 Hz tone burst that was shaped by a rectangular gating function and generated digitally at a 44.1 kHz sampling rate. The stimulus was presented to the implanted ear via an insert earphone at a 10 Hz stimulation rate. The level of the acoustic stimulus was varied from MCL down to visual ECochG threshold in 5–10 dB steps. ECochG responses were recorded using both positive and negative leading tone burst stimuli. For this study, only the ECochG response at MCL was examined. Electrode 22 (the most apical intracochlear electrode) was used as the recording electrode. Recording sampling rate was 20 kHz. Each response consisted of an average of 200 to 400 sweeps. Contamination due to system artifacts were minimized by obtaining an ECochG response when the acoustic probe was not placed in ear canal, but continued to deliver an acoustic stimulus at the highest test level. This “no stimulus” recording of system artifact was subtracted from the ECochG recordings.

Responses recorded using initially positive and negative polarities were stored separately and analyzed in the frequency domain using a Fast Fourier Transform (FFT). The resolution of the FFT was 55.33 Hz/bin. These ECochG responses likely reflect activity generated at both the hair cell (i.e., cochlear microphonic) and the auditory nerve (i.e., auditory nerve neurophonic). The data from Abbas et al. (2017) were reanalyzed using different techniques. The magnitudes of the FFT responses recorded at the frequency corresponding to the first, second, and third harmonics of the tone burst were measured and were considered significant if the amplitude exceeded the noise plus three standard deviations. The noise and its standard deviation were calculated from 6 bins, 3 on each side of all harmonics, starting 2 bins away from the peak. Magnitude of ECochG was calculated as the sum of the magnitude of FFT responses at all significant harmonics in each polarity. For this study, the average of the magnitude of ECochG in each polarity was used for correlational analysis.

### Speech perception measures

Two different measures of speech perception were obtained from the clinical records of each subject. Speech perception in quiet was measured using the CNC monosyllabic word test (Peterson and Lehiste, 1962). Speech perception in noise was assessed using the AzBio sentence test with the sentences presented at a +5 dB signal-to-noise ratio (SNR) (Spahr et al., 2012). The noise used for the AzBio sentence test was a 10-talker babble. For both tests, the speech signal was presented at 60 dBA via a loudspeaker located 1 meter away from the subject at 0 degrees azimuth. Noise was also presented from the same loudspeaker for the AzBio test. The CNC word test consists of 50 words in each list, and the AzBio sentence test is composed of 20 sentences in each list. Two lists were used for both tests. Results were reported in percentage of the total number of words correct.

Speech perception data obtained in the E alone (implant alone) and A+E (implant and ipsilateral hearing aid) listening conditions were extracted from the medical charts. To assess speech performance in the E alone mode, both ipsilateral and contralateral ear canals were occluded with foam earplugs and earmuffs. For performance in the A+E mode, only the contralateral ear canal was occluded. Pilot data collected from two normal hearing listeners revealed that use of plugs and muffs resulted in 25 to 40 dB of attenuation for frequencies between 125 and 1,000 Hz and 25 dB of attenuation for speech reception thresholds in the sound field. Clearly, we cannot argue that contribution from the non-test ear was eliminated; however, it should have been minimized based on these attenuation rates. Finally, we also calculated a metric we refer to as acoustic gain (A gain). A gain was computed by subtracting the E alone score from the A+E score. In theory, this subtracted response should reflect the benefit individual study participants receive from the use of their residual low frequency acoustic hearing.

## Results

### Electrophysiologic measures

**Figure 2:**
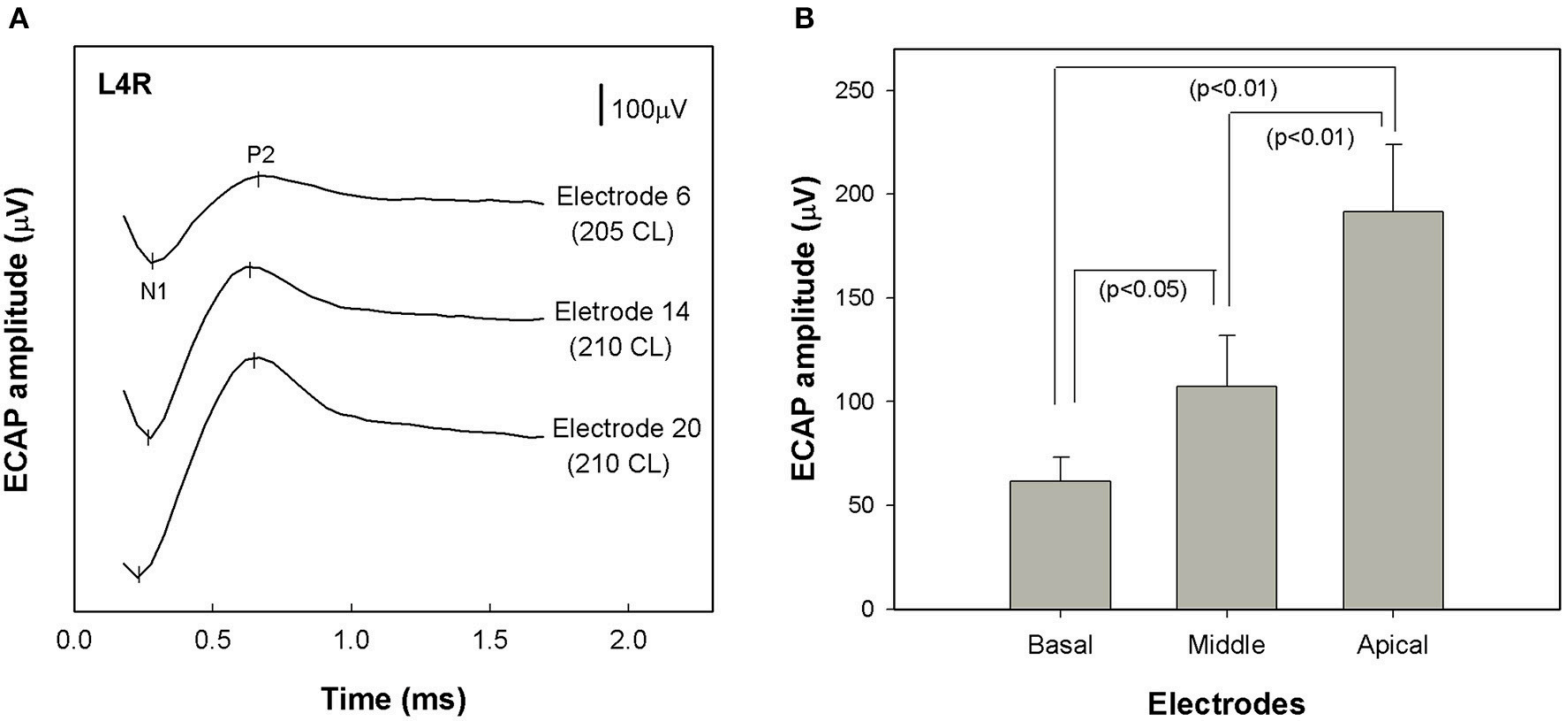
**(A)** ECAP waveforms recorded from a single study participant (L4R) at three different stimulation sites: electrode 6 (basal), electrode 14 (middle), and electrode 20 (apical). Each waveform was recorded at the maximum acceptable loudness level. Stimulation level is specified in clinical programming units (CL). **(B)** Comparison of group mean ECAP amplitudes across stimulating electrode sites (basal, middle and apical) (*n* = 24). Error bars indicate ± 1 standard error around the mean. ECAP stands for electrically evoked compound action potential.

ECAP recordings were obtained for 24 of the 25 study participants (96%). We attempted, but failed to record an ECAP for one participant. ECAP thresholds were possibly higher than MCL in this case. Figure 2A shows typical ECAP waveforms measured using stimulation of electrode 6 (basal), 14 (middle) and 20 (apical) for subject L4R. ECAP amplitude decreased as the stimulating electrode was changed from an apical to a more basal electrode.

Figure 2B shows the range of ECAP amplitudes recorded at each of the three stimulation sites. A repeated measures analysis of variance (ANOVA) was performed using stimulation site (apical, middle, and basal) as the within-subjects variable. The analysis revealed a significant effect of stimulation site [*F*_(1.482, 24)_ = 19.461, *p* < 0.01]. *Post-hoc* tests indicated that ECAP amplitudes became progressively larger as the stimulating electrode was moved toward the apex of the electrode array. Specifically, the ECAP amplitudes recorded with stimulation near the middle of the array were significantly greater than those recorded using a more basal stimulation site (*p* < 0.05) and were significantly smaller than those recorded using more apical stimulation (*p* < 0.01).

**Figure 3:**
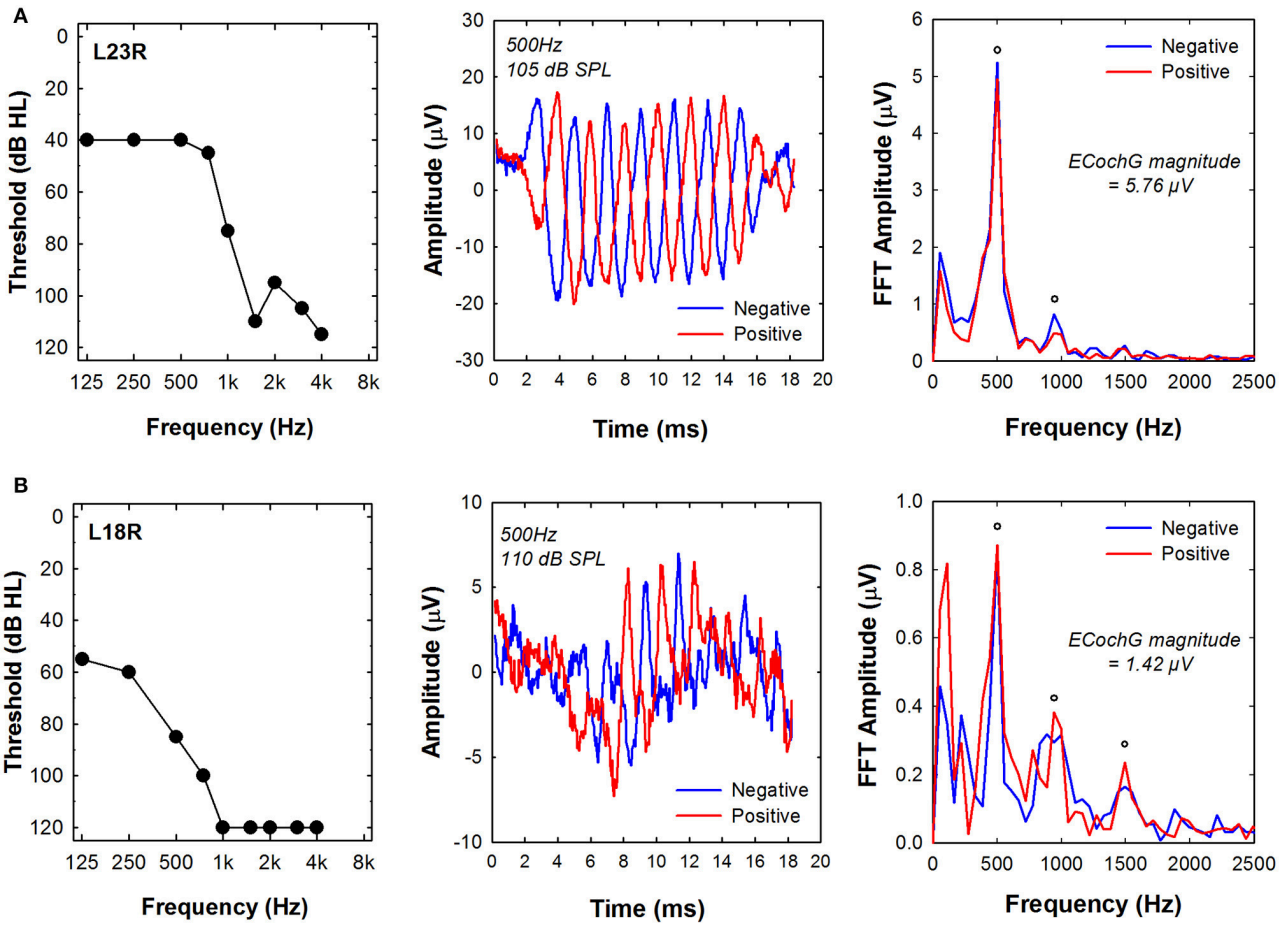
**Examples of ECochG responses recorded using a 500 Hz tone burst from two subjects, (A)** L23R and **(B)** L18R. Left panels show the audiogram measured at the time of testing. Center panels show the time waveforms in response to positive and negative leading tone bursts. Right panels show the results of a FFT of the time waveforms. The circles indicate frequencies where the energy at the harmonic frequency was significantly above the noise floor of the measurement system. Note the different amplitude scales used for these two subjects. ECochG indicates electrocochleography; FFT, Fast Fourier Transform.

ECochG responses were recorded using 500 Hz tone bursts from all of the study participants. Figure 3 shows example recordings obtained from two different subjects (L23R, L18R). The two panels on the left side of Figure 3 show the pure tone audiogram for the implanted ear measured at the test session. 500 Hz audiometric thresholds were 40 dB HL for subject L23R and 85 dB HL for subject L18R. The center panels show ECochG waveforms recorded using 500 Hz tone bursts that were presented at MCL and in both polarities for each of the two subjects. The panels on the right side of Figure 3 show the results of FFT analysis of ECochG recordings. Clear peaks in the FFT are apparent at 500 Hz and 1,000 Hz for subject L23R whose data is shown in the top row. For subject L18R, clear peaks in the FFT were evident at 500, 1,000, and 1,500 Hz. The frequencies correspond to the first, second and third harmonics of the 500 Hz stimulus. The circles indicate FFT responses where the specific harmonic was significantly above the noise floor of the measurement system. ECochG magnitude was calculated by averaging the sum of the magnitude of responses at all significant harmonics in each polarity. These values are indicated on the figure. Note that the magnitude of the ECochG response is larger for the subject with more residual hearing (L23R).

### Speech perception measures

**Figure 4:**
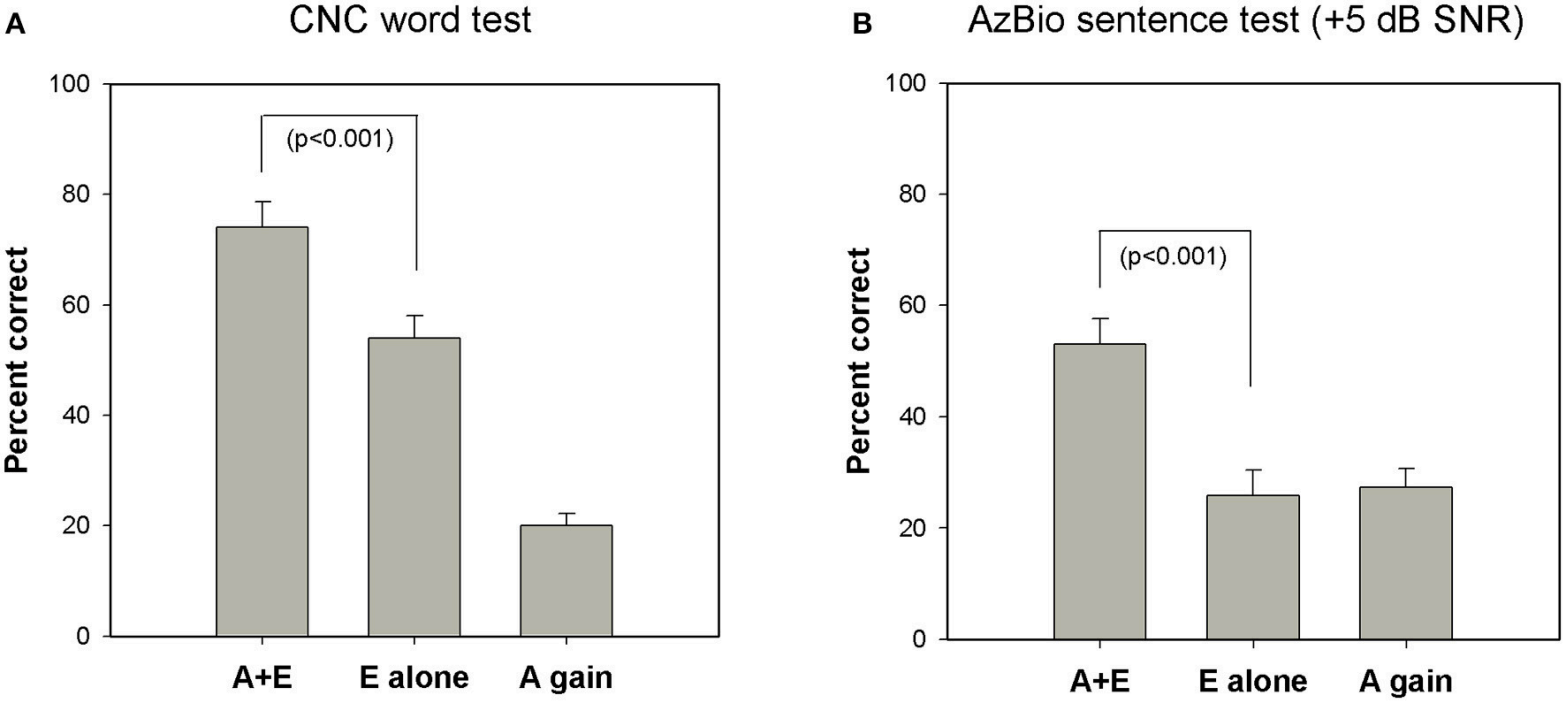
**Group mean speech perception scores in A+E, E alone, and A gain condition on (A)** CNC word test in quiet (*n* = 23) and **(B)** AzBio sentence test at a +5 dB SNR (*n* = 22). Error bars are standard errors of means. CNC indicates consonant-nucleus-consonant; SNR, signal-to-noise ratio; A, acoustic; E, electric.

Figure 4 shows the effect of listening mode on speech perception measured in quiet (CNC word test) and in background noise (AzBio sentence test at +5 dB SNR). CNC scores were not available for two subjects and AzBio test results were not measured for three subjects in the E alone modes. For the CNC word test, mean scores were 74% in A+E mode and 54% in E alone mode. For the AzBio sentences test in noise, the mean scores were 53% in A+E condition and 26% in E alone condition. Performance in the A+E mode was significantly better than in E alone mode for both tests as shown by a paired samples *t*-test [CNC: *t*_(22)_ = 9.12, *p* < 0.001; AzBio: *t*_(21)_ = 8.23, *p* < 0.001].

This figure also shows that average performance in the E alone condition was greater when the task involved perception of speech in quiet (CNC test) compared to when the task required perception of speech in background noise (AzBio test). However, the benefit provided by having access to acoustic sound (A gain) is greater for speech perception in noise (AzBio test) compared to speech perception in quiet (CNC test). That is, the difference between the E alone and A gain scores is greater for the CNC test than for the AzBio test. While this may be due to differences in test materials (words vs. sentences), we suggest that it may also reflect that for speech stimuli presented in background noise, greater reliance on the acoustic signal is required for better performance.

In order to quantify the contribution of electric and acoustic stimulation to performance in the A+E listening mode, we computed two ratios for each subject. One ratio compared the speech perception score obtained in the E-alone condition to the A+E condition (E-alone/A+E). A second ratio compared speech perception score obtained using only acoustic stimulation (A gain) to the score obtained in the A+E listening mode (A gain/A+E). Paired t-tests revealed that the ratio of E alone/A+E was significantly larger for the CNC test compared to the AzBio test [*t*_(21)_ = 6.75, *p* < 0.001]. A similar analysis was performed comparing the ratio of A gain/A+E on the two speech perception tasks. Paired *t*-tests showed that the ratio of A gain/A+E was significantly larger for the AzBio test in noise than for the CNC test in quiet [*t*_(21)_ = 4.59, *p* < 0.001]. (Note that the sum of the E alone ratio and the A gain ratio will be 1, thus this analysis is complementary). We interpret these data to suggest that electric hearing may contribute more to the benefits of hybrid listening in quiet environments, while residual acoustic hearing is an important factor that may play a larger role in determining outcomes in noisy listening conditions.

**Figure 5:**
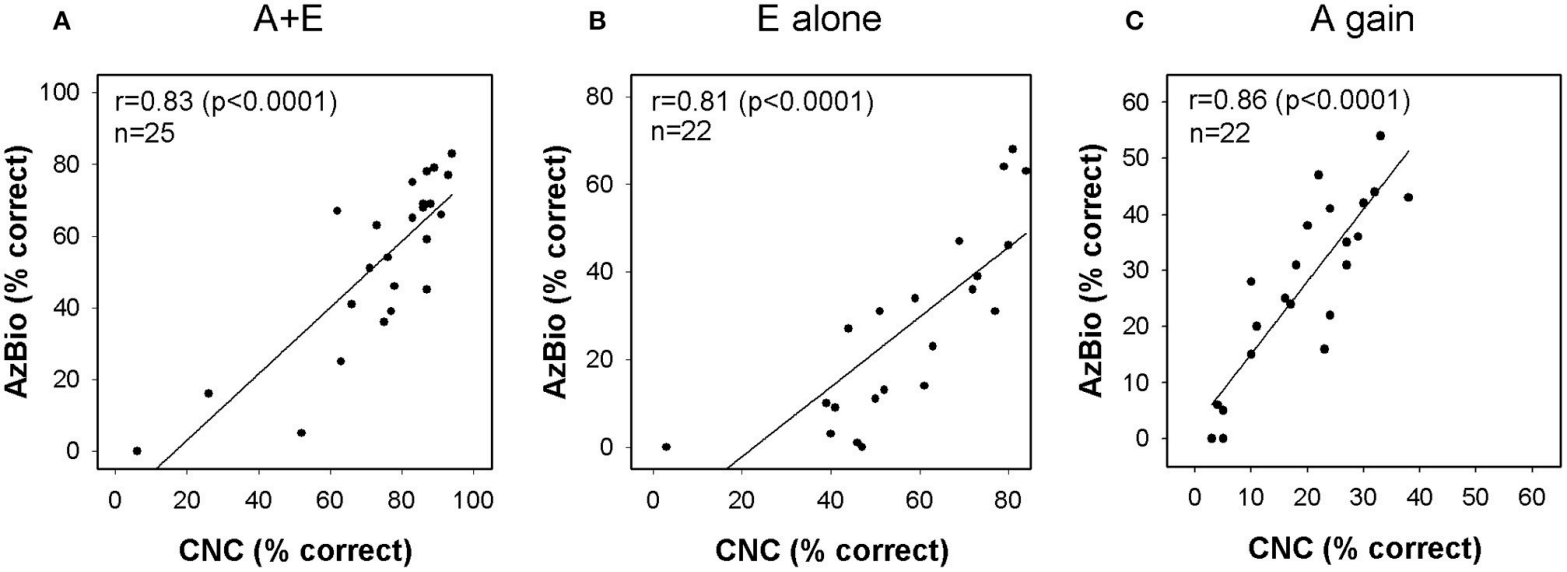
**Relationship between CNC and AzBio performance in (A)** A+E, **(B)** E alone, and **(C)** A gain conditions. Each column plots the AzBio sentence scores as a function of CNC word scores. CNC indicates consonant-nucleus-consonant; A, acoustic; E, electric.

Figure 5 shows correlations between performance on CNC word lists presented in quiet and AzBio sentences presented in noise. Linear regression analysis revealed significant correlation in scores for A+E (*r* = 0.83, *p* < 0.0001, *n* = 25), E alone (*r* = 0.81, *p* < 0.0001, *n* = 22) and A gain (*r* = 0.85, *p* < 0.0001, *n* = 22) conditions. Subjects who perform better on one speech perception test are likely to perform better on another measure of speech perception.

### Correlations between electrophysiologic measures and performance

The primary goal of this study was to characterize the relationship between electrically and acoustically evoked peripheral electrophysiologic measures and performance on speech perception tests in a representative group of Nucleus Hybrid L24 CI users. Hybrid CI users perceive high-frequency portions of the acoustic signal via electric hearing. Low frequency information in the acoustic signal is amplified and transmitted acoustically. Therefore, ECAP responses to electrical stimulation were compared to performance in E alone condition and ECochG responses to acoustic stimulation were compared to A gain. ECAP and ECochG responses were also compared to performance in A+E condition.

**Figure 6:**
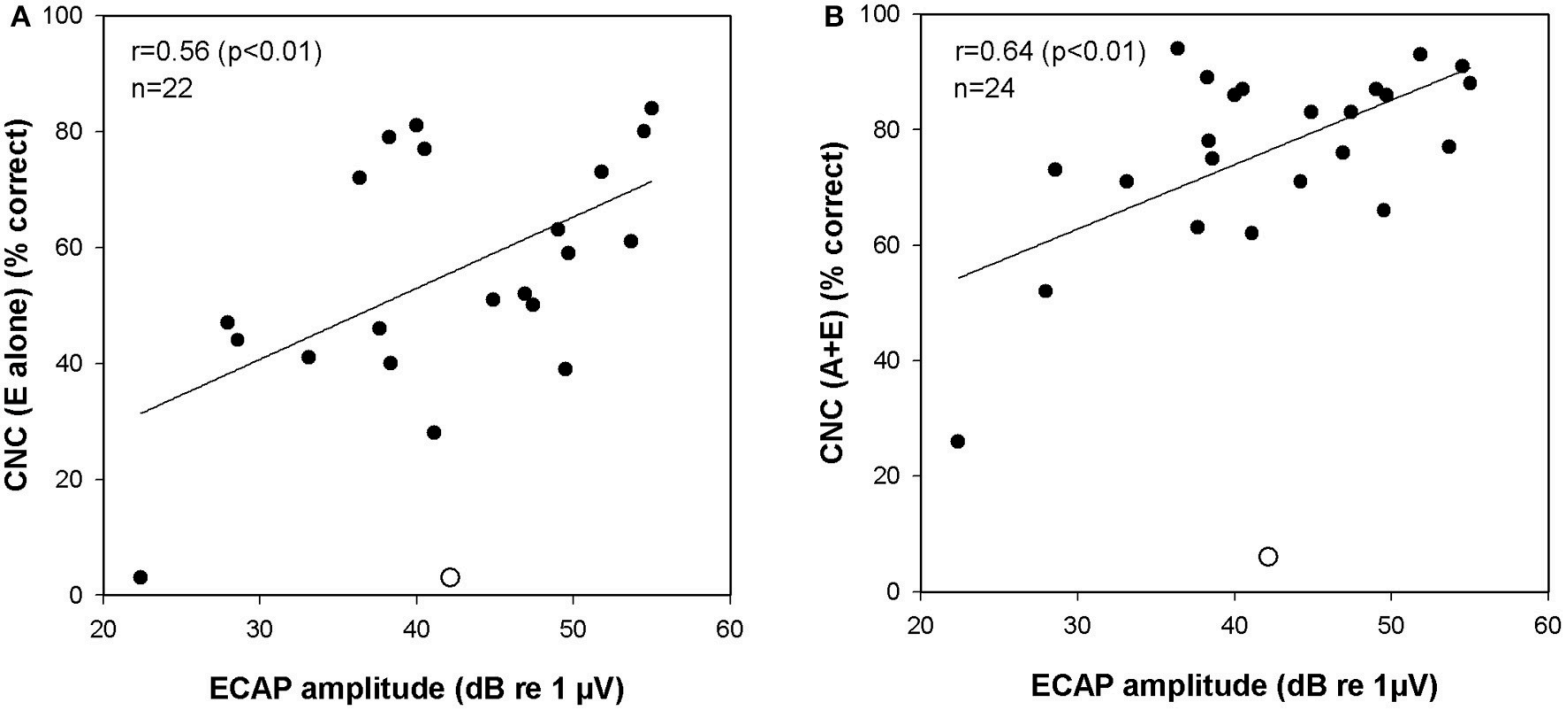
**(A)** Correlation between ECAP amplitudes and CNC scores (E alone). **(B)** Correlation between ECAP amplitudes and CNC scores (A+E). One data point was identified as an outlier (studentized residual > 2.0). This data point is marked with an open circle and was excluded from correlation analysis. ECAP indicates electrically evoked compound action potential; CNC, consonant-nucleus-consonant; A, acoustic; E, electric.

We hypothesized that performance on speech tests, particularly when testing is done in the E alone condition, would correlate with electrically evoked responses. We found that the correlation between the amplitude of the ECAP recorded using stimulation of the most apical electrode in the intracochlear array and performance on the CNC word list administered in the E alone mode was, in fact, statistically significant (*r* = 0.56, *p* < 0.01). Figure 6A is a scatterplot that illustrates this relationship. No significant correlation between the ECAP amplitudes recorded from the middle or basal electrode and CNC performance were revealed nor were there significant correlations between ECAP amplitude and performance on the more challenging AzBio test when administered in the E alone mode.

We also hypothesized that speech perception in the A gain condition would be related to the acoustically evoked ECochG responses. We assumed that subjects who benefited most from the use of their residual acoustic hearing have more robust ECochG responses to a low frequency tone burst. However, no statistically significant correlations were found between the magnitude of the ECochG recorded using a 500 Hz tone burst and performance on either the CNC word lists or on the AzBio test in the A gain condition.

While correlations between the ECAP or ECochG and performance in the E alone and A gain conditions are informative, more important are correlations between these peripheral measures of auditory function and performance in the A+E listening mode. This is the condition where the subjects are most practiced and, from a clinical perspective, it is the most relevant test mode. The amplitude of the ECAP response recorded using stimulation of the most apical electrode was found to be significantly correlated with performance on the CNC word list when speech perception was measured in the A+E listening mode (*r* = 0.64, *p* < 0.01). Figure 6B is a scatterplot that illustrates this relationship. Significant correlations between the apical ECAP amplitude and performance on the AzBio test were not observed nor were significant correlations between the ECAP amplitudes recorded from middle or basal electrodes and speech performance revealed. The ECochG magnitudes were also compared to performance in A+E listening mode. However, there were no significant correlations between the ECochG magnitude and performance on CNC or AzBio tests.

Table 2 shows the summary of correlations between ECAP (recorded from an apical electrode) and ECochG responses to speech perception scores. No significant correlations were found for middle and basal electrodes; thus, for brevity, they were not included in Table 2.

**Table 2:** **Summary of correlation between peripheral electrophysiologic measures and speech performance**.

**EP measure**	**Speech perception measure**	***n***	**Pearson's *r***	***p***
ECAP	CNC word test			
	E alone	22^†^	0.56	0.009^*^
	A+E	24^†^	0.64	0.001^*^
	AzBio sentence test			
	E alone	21	0.35	0.118
	A+E	24	0.38	0.070
ECochG	CNC word test			
	A gain	23	0.06	0.780
	A+E	25	0.23	0.267
	AzBio sentence test			
	A gain	22	0.18	0.412
	A+E	25	0.20	0.343

*Note that ECAPs listed in this table were measured from apical electrodes*.

† *One data identified as an outlier was excluded from correlation analysis*.

* *p < 0.05*.

*EP indicates electrophysiologic; ECAP, electrically evoked compound action potential; ECochG, electrocochleography; CNC, consonant-nucleus-consonant; A, acoustic; E, electric*.

We know that Hybrid CI users enjoy improved hearing in noise rather than in quiet relative to standard, long electrode CI users, likely due to the residual low frequency acoustic hearing (Turner et al., 2004; Gantz et al., 2009). In order to further investigate the relationship between the acoustically evoked ECochG measures and performance with the Hybrid implant, we computed a ratio of performance on speech perception in noise (AzBio test) relative to their ability to understand speech in quiet (CNC test). This was calculated as

$$\begin{array}{l} A_{gain}\;Ratio=\;\frac{A_{gain\;\left(Noise\right)}}{A_{gain\;\left(Quiet\right)}}=\frac{AzBio_{A+E}-AzBio_{E\;alone}}{CNC_{A+E}-CNC_{E\;alone}} \end{array}$$

**Figure 7:**
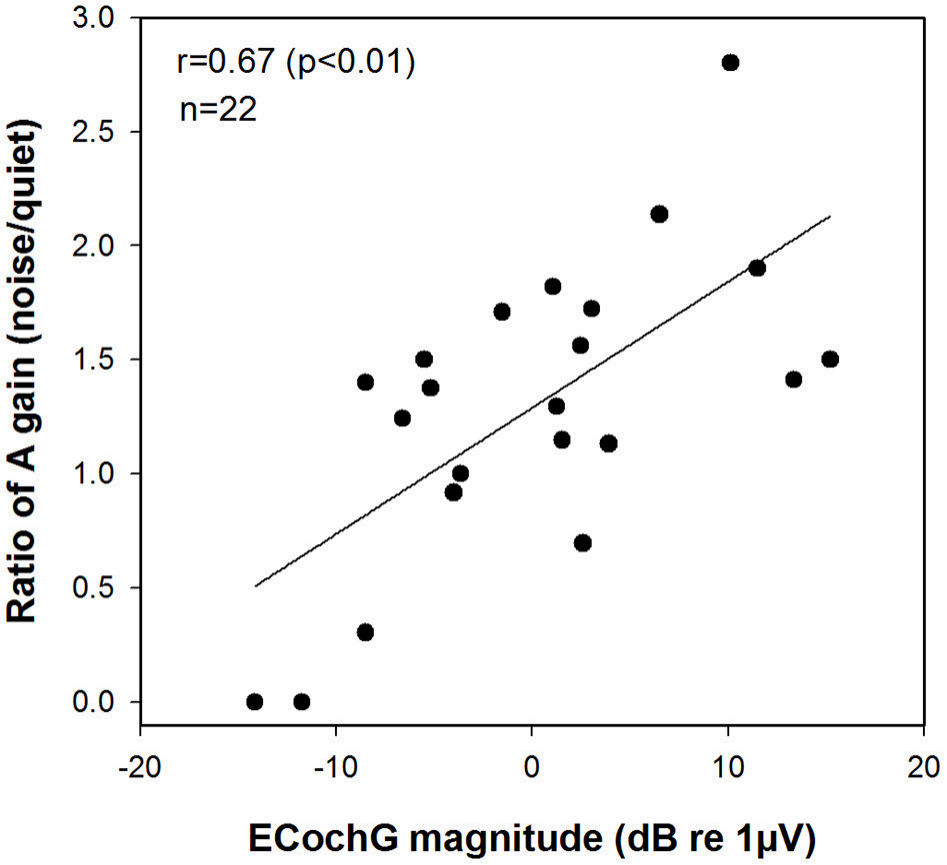
**Correlation between the magnitude of the ECochG response to a 500 Hz tone burst and the ratio of A gain scores on speech perception test in noise relative to quiet (AzBio/CNC)**. ECochG indicates electrocochleography; CNC, consonant-nucleus-consonant; A, acoustic.

We focused on the derived A gain scores, reasoning that these measures are the ones likely to be most sensitive to the status of the cochlea. Figure 7 shows the results we observed when we made this comparison. The magnitude of the ECochG response to a 500 Hz tone burst was found to be correlated with speech perception as characterized using this ratio (A gain) of two different speech tests (*r* = 0.67, *p* < 0.01).

Lastly, we attempted a multiple regression analyses to look at the predictive values of both ECAP and ECochG metrics on speech perception scores. We performed it twice—one on CNC scores and once on AzBio scores. The analysis revealed that there was a significant correlation between the maximum amplitude of the ECAP (apical electrode) and performance of A+E (*F* = 5.9851, *p* < 0.05) on CNC words test. However, there was no significant correlation between ECochG and A+E scores on CNC words test nor was a statistically significant correlation found between performance on the AzBio test and either ECAP or ECochG magnitude.

## Discussion

The primary goal of this study was to determine the extent to which ECAPs recorded using electrical stimulation and ECochG responses recorded using acoustic stimulation were related with speech perception in Nucleus Hybrid L24 CI users. The reasoning is that ECAPs would reflect activity along different points of the electrode array, which is seated basally in the cochlea. ECochGs would reflect activity along more apical regions of the cochlea. Using both metrics may more fully characterize the status of the cochlea. To our knowledge, the present study is the first to investigate the relationship between post-operative peripheral electrophysiologic measures (specifically acoustically evoked potentials) and speech performance in hearing preservation implants. Prior studies have only demonstrated the feasibility of making such recordings (Dalbert et al., 2015; Abbas et al., 2017; Koka et al., 2017) but the clinical applicability of these measures need to be addressed beyond their ability to predict audiometric thresholds. It also extends previous studies that have found correlations between intraoperative ECochG measures in CI users and speech outcomes (Fitzpatrick et al., 2013; Formeister et al., 2015).

The Hybrid L24 CI does not extend along the full length of the cochlea. Additionally, neural survival is not likely to be uniform along the cochlear partition. Indeed, audiometric thresholds are better for low frequencies and poorer for high frequencies in our Hybrid CI users (see Figure 1). This observation suggests that neural survival is likely to be better closer to the apex than the base of the cochlea. ECAP recordings primarily reflect activity of neurons located along the relatively basal region of the cochlea (given that electrode arrays do not span the entire cochlea). We hypothesized that the position of the electrode array and the configuration of the hearing loss would result in larger ECAP responses for apical electrodes. That is, in fact, what we found (see Figure 2). ECAP amplitudes recorded from more apical electrodes are significantly larger than those recorded from more basal electrodes.

We assume that larger ECAP amplitudes may reflect better neural survival and that, in turn, may lead to better performance on tests of speech perception, particularly when the listening mode is E alone. Our results showed that the ECAP amplitudes recorded from apical electrodes are significantly correlated with speech perception as measured using CNC word tests (see Figure 6A). This finding is consistent with Kim et al. (2010) in which the slope of the ECAP amplitude growth function obtained from Nucleus Hybrid S8 (RE) and Standard CI24RE CI users were significantly correlated with speech perception. The slope metric was used as a marker of neural survival, similar to animal studies (Smith and Simmons, 1983; Hall, 1990; Miller et al., 1994; Prado-Guitierrez et al., 2006). The L24 Hybrid also has the same receiver-stimulator as the Hybrid S8 (RE) and standard 24RE, so results can be compared across devices. However, correlations are not often seen between electrophysiologic measures and speech perception in older devices (Abbas and Brown, 1991; Brown et al., 1995; for review see Van Eijl et al., 2017). Studies comparing post-mortem spiral ganglion neuron counts to speech outcomes don't often see correlations either (e.g., Nadol et al., 2001; Khan et al., 2005; Fayad and Linthicum, 2006; however, see Seyyedi et al., 2014). Mixed findings are not surprising; the ECAP is a peripheral response while speech perception requires peripheral and central processes, as well as cognitive resources. Both peripheral and central measures may be needed to increase the predictive power of electrophysiologic measures (Scheperle and Abbas, 2015). ECAP amplitudes recorded from middle or basal electrodes did not show correlations with CNC scores. ECAP responses were not as robust for these electrodes, possibly due to less neural survival, which may have precluded meaningful correlational analysis with speech outcomes.

No correlation between ECAP amplitude and performance on the AzBio sentence test in noise was obtained regardless of the stimulating electrode used. It may be that ECAP amplitudes do not reflect the spectral/spatial resolution needed for speech perception in noise. CI users require more electrodes for speech perception in noise relative to quiet, since more electrodes potentially provide better spectral/spatial resolution (Friesen et al., 2001). Experiments using vocoded speech have shown that only a few spectral bands are needed for adequate speech recognition in quiet (Shannon et al., 1995; Xu and Zheng, 2007), but more bands are needed for speech recognition in noise (Qin and Oxenham, 2003; Xu and Zheng, 2007), reflecting the contribution of increased spectral resolution to speech recognition in noise. Spatial resolution can be inferred from channel interaction measures made using ECAPs (Abbas et al., 2004), with a recent study demonstrating correlations between channel interaction measures and speech perception in noise (Scheperle and Abbas, 2015).

While ECAPs provide a measure of the response of the auditory nerve to electrical stimulation, ECochG responses include contributions from both cochlear hair cells and from the auditory nerve following acoustic stimulation. We know that use of a Hybrid CI improves speech understanding in part because it allows the listener to use his/her acoustic hearing to perceive low frequency cues in an acoustic signal and to use the electrical signal provided by the CI to perceive high frequency information (Turner et al., 2004; Ching, 2005; Brown and Bacon, 2009; Zhang et al., 2010). Our results also showed that performance in the A+E listening mode was significantly better than in E alone mode for both CNC and AzBio tests (see Figure 4) and demonstrate that preserving residual acoustic hearing was beneficial for our population of study participants. The ECochG recordings obtained using a 500 Hz tone burst provide a measure of how the auditory periphery responds to a low frequency acoustic stimulus. Here we suggest that ECochG recordings may provide a metric that reflects the overall “health” of at least the apical portion of the cochlea. We hypothesize that the ECochG magnitude measures might be more strongly correlated with A gain speech perception scores rather than results of tests conducted in the A+E or E alone listening modes. However, we found no significant correlations between ECochG responses and A gain scores on CNC words test nor on the AzBio sentences test. The lack of a correlation may be because A gain scores are not a direct measure of speech perception abilities in the A alone condition. We treated these measures as additive, assuming acoustic only scores plus electric only scores equals A+E score, which is not necessarily the case. Gifford et al. (2008a) tested S8 hybrid patients on word recognition in acoustic only, electric only, and A+E listening modes. None of those patients had A+E scores that were equal to A only + E only score. It seems equally likely, however, that in addition to cochlear health, other factors such as patient demographics, cognitive ability, and genetic variants may affect performance on speech perception tests, increase variance in our measures and reduce the correlations evident in this study (Lazard et al., 2012; Blamey et al., 2013; Holden et al., 2013; Shearer et al., 2017).

Compared to individuals who use standard CIs, Hybrid CI users perform better on tests of speech perception in background noise than in quiet. Several investigators have attributed this to residual low frequency acoustic hearing providing significant benefits—particularly when the task involves understanding speech in background noise (Turner et al., 2004, 2008b; Gantz et al., 2009; Zhang et al., 2010; Carroll et al., 2011). For example, Turner et al. (2004, 2008b) showed that Hybrid CI users outperformed standard CI users on tests of speech perception in background noise, even though these two groups had equivalent levels of speech perception in quiet. This advantage is primarily a result of the better frequency resolution provided by the residual acoustic hearing (Qin and Oxenham, 2003; Turner et al., 2004). We expect, therefore, that the benefits enjoyed by Hybrid CI users would be most evident in situations, such as speech perception in noise, where frequency resolution is important. Our results also suggested that acoustic hearing (A gain) plays a larger role in determining how well speech is perceived in noise (AzBio test) compared to quiet (CNC test), even though performance is better in the A+E mode compared to the E alone mode on both CNC and AzBio tests (see Figure 4). This is in general agreement with findings from other studies (Kiefer et al., 2005; Zhang et al., 2010). Therefore, we assumed that the benefits of acoustic hearing in noise relative to in quiet may be predicted by our ECochG data that has been proposed to serve as a measure of cochlear health. We did find that ECochG magnitude was significantly correlated with the ratio of the AzBio score and the CNC score when both were collected in the A gain condition (see Figure 7). This finding is consistent with an assumption that the magnitude of the ECochG response evoked using a 500 Hz tone burst may serve as an index to overall cochlear health at the apical region and at least partially explain benefit provided to the listener by their residual low frequency acoustic hearing. It could be argued, however, that the composite metric was made by using two different tests and may not accurately reflect the benefit of A gain in noisy situations. The CNC word test and the AzBio sentence test have differing cues, such as lexical, semantic, context, and acoustic cues, and could have differing distributions of speech scores amongst the patient population. However, Gifford et al. (2008b) reported a significant correlation between performance on CNC word lists and AzBio sentences presented in quiet (*r* = 0.85, *p* < 0.0001). Moreover, our results also revealed strong correlations between performance on the CNC word list presented in quiet and AzBio sentences presented in noise for each condition (A+E, E alone, and A gain) (see Figure 5).

This study also explored the correlation between the ECAP or ECochG and speech perception measured in the A+E listening mode. The ECAP amplitudes were significantly correlated with performance on CNC test (see Figure 6B), but not correlated with performance on AzBio test. We found that the ratios of E alone score to A gain score were approximately 7:3 and 5:5 for CNC word test presented in quiet and AzBio sentence test presented in noise, respectively (see Figure 4). That is, the high frequency portions of the speech signal conveyed electrically made a dominant contribution to speech perception in quiet as described in other studies (Kiefer et al., 2005; Turner et al., 2008a). This was not the case for speech perception in noise. Our results show that electrically evoked neural responses seems to be more predictive of performance when the task does not include background noise (e.g., the CNC test) and when testing is conducted in the A+E listening condition.

We assumed that the acoustically evoked ECochG magnitudes might serve as an index of overall cochlear health and as such might predict performance on speech perception tests. There was a tendency for magnitude of the ECochG responses evoked using the 500 Hz tone burst to be correlated with audiometric thresholds (e.g., see Figure 3). However, the ECochG measures we recorded were not correlated with outcome on either speech test when testing was conducted in the A+E listening modes. For example, despite differences in residual acoustic hearing and ECochG magnitude, speech perception results were similar for both subjects (L23R and L18R) whose data are shown in Figure 3. These results stand in contrast to data reported by Fitzpatrick et al. (2013) and Formeister et al. (2015) showing significant correlations between physiologic measures of “total cochlear response” (representing a sum of responses using 250 to 4,000 Hz tone bursts recorded using a round window electrode prior to insertion of the electrode array) and postoperative speech perception. In this study, we recorded ECochG responses from an intracochlear electrode rather than from the round window. Our recordings were also obtained post-operatively rather than prior to the insertion of the electrode array into the cochlea. We reasoned that an intracochlear recording electrode would be closer to the cochlear hair cells and auditory neurons and as such, could be more reflective of cochlear health than similar measures obtained from the round window. Therefore, we would have expected to find a better correlation between a postoperative electrophysiological measures and speech perception than had been reported previously. That was not the case. However, we used only one tone burst frequency to evoke the ECochG response, while Fitzpatrick et al. (2013) and Formeister et al. (2015) used several tone burst frequencies. Our results may, therefore, represent a measure of cochlear health from a more restricted region on the cochlea. We also assumed that insertion of the electrode array into the cochlea would be likely to affect cochlear function and as a result, post-implant measures would accurately predict outcome with a Hybrid CI than pre-operative measures. Our assumption may not have been valid. Animal studies show that it is possible to insert the electrode array into the cochlea and only transiently affect the ECochG (DeMason et al., 2012). If so, the impact on speech perception may not be significant and could also explain the difference between our results and those of Fitzpatrick et al. (2013) and Formeister et al. (2015). Perhaps recording the ECochG pre- and post-insertion could provide a more complete picture of overall cochlear health and the combination of those data with ECAP recordings may improve our ability to predict speech perception outcomes.

The results of the present study suggest that peripheral electrophysiologic responses to both acoustic and electric stimuli may be important to fully characterize the status of the cochlea for an individual Hybrid CI user and may be required to improve our ability to predict speech perception outcomes. While we did find correlations between ECAP or ECochG measures and speech perception, we acknowledge that there were fewer significant correlations than non-significant correlations and one might reasonably argue that the few significant correlations that were observed arose out of chance. A well-controlled prospective study design is needed to address the limitations of the current study.

This study has also some limitations due to the retrospective nature of the design. We tried to use similar metrics for both ECAP and ECochG data and focused on the amplitudes. Our ECAP growth functions had more data points, allowing us to visually determine the threshold and calculate slope. However, experimental limitations, as outlined in Abbas et al. (2017), prevented us from collecting many data points for a finely detailed growth function for ECochG responses. The ECochG thresholds from that study were calculated based on linear regression fits to the ECochG amplitude growth function rather than visual detection thresholds. Thus, we wanted to avoid using two different methodologies. Future prospective studies should collect ECochG amplitude growth functions with multiple levels, as well as at multiple frequencies. This would allow the use of visual detection thresholds, slopes, and amplitudes across different frequencies and levels to more fully characterize acoustic responses. Future studies can also use measurement and analysis techniques to emphasize responses from the hair cell and from the auditory nerve and correlate this to outcomes. Such studies might not only result in more accurate prediction of overall outcome with Hybrid CIs than have been available previously but also provide important clues as to the source of the cross-subject variance routinely observed in CI populations.

While Hybrid CI users currently are a small section of the CI population, there is increasingly more emphasis on the use of soft surgical techniques and electrode designs that may help reduce cochlear trauma. Multicenter trials have demonstrated hearing preservation is possible with both short (Gantz et al., 2009; Lenarz et al., 2013; Roland et al., 2016) and long electrode arrays (Santa Maria et al., 2013; Van Abel et al., 2015; Hunter et al., 2016). Our results show the relative contributions of acoustic and electric hearing to speech perception in quiet and noise. We would argue that if preservation of residual acoustic hearing in the implanted ear remains an important goal both for surgeons and CI manufacturers, methods to evaluate the contributions of residual acoustic hearing and electrical stimulation to speech perception will be necessary.

## Conclusions

ECAPs reflect response of auditory neurons across the electrode array, seated at the relatively basal regions of the cochlea. ECochG responses provide a way to assess the response of the cochlear hair cells and auditory nerve for neurons innervating more apical regions of the cochlear partition. Both can be recorded from Hybrid L24 CI users. The results of this study suggest that outcomes with a Hybrid CI on tests of speech perception in quiet and/or in noise can be more accurately characterized by using both ECAP (recorded from an apical electrode) and ECochG measures rather than either metric alone.

## Author contributions

JK, VT, PA, and CB: Substantial contributions to the conception or design of the work; Substantial contribution to the acquisition, analysis, or interpretation of data for the work; Drafting the work and revising it critically for important intellectual content; Final approval of the version to be published; Agreement to be accountable for all aspects of the work in ensuring that questions related to the accuracy or integrity of any part of the work are appropriately investigated and resolved.

## Funding

This work was supported by grants from the NIH/NIDCD (P50 DC000242) and funding for a “research year” provided by Inje University in 2015.

### Conflict of interest statement

The authors declare that the research was conducted in the absence of any commercial or financial relationships that could be construed as a potential conflict of interest. The reviewer AJO and handling Editor declared their shared affiliation, and the handling Editor states that the process nevertheless met the standards of a fair and objective review.
